# Molecular imaging of pulmonary diseases

**DOI:** 10.1186/s12931-018-0716-0

**Published:** 2018-01-24

**Authors:** Julien Dimastromatteo, Eric J. Charles, Victor E. Laubach

**Affiliations:** 1 Department of Biomedical Engineering, University of Virginia, Charlottesville, VA USA; 2 Department of Surgery, University of Virginia, P.O. Box 801359, Charlottesville, VA 22908 USA

**Keywords:** Molecular imaging, Pulmonary disease, SPECT, PET

## Abstract

Imaging holds an important role in the diagnosis of lung diseases. Along with clinical tests, noninvasive imaging techniques provide complementary and valuable information that enables a complete differential diagnosis. Various novel molecular imaging tools are currently under investigation aimed toward achieving a better understanding of lung disease physiopathology as well as early detection and accurate diagnosis leading to targeted treatment. Recent research on molecular imaging methods that may permit differentiation of the cellular and molecular components of pulmonary disease and monitoring of immune activation are detailed in this review. The application of molecular imaging to lung disease is currently in its early stage, especially compared to other organs or tissues, but future studies will undoubtedly reveal useful pulmonary imaging probes and imaging modalities.

## Background

Imaging holds an important complementary role in the diagnosis of lung diseases. Along with clinical tests, imaging techniques provide valuable information that enables a complete differential diagnosis. Often, lung disease symptoms are similar and begin to be discriminative only at a late stage when prognosis of the ultimately named pathology is already less favorable. Currently, chest X-ray, computed tomography (CT) and magnetic resonance imaging (MRI) are conventional imaging modalities first prescribed after patient examination. With high spatial resolution, these modalities provide anatomical and structural information of the lungs, but in order to differentiate between lung diseases with overlapping pathophysiology or to enable diagnosis at a very early stage, a more accurate approach is necessary. To this extent, the emerging field of molecular imaging is of great relevance. Molecular imaging is a field at the intersection of molecular biology and conventional medical imaging [1]. Unconventional imaging modalities such as single photon emission computed tomography (SPECT) and positron emission tomography (PET) exhibit high sensitivity and resolution to provide information at a molecular level. Table 1 lists the general characteristics as well as the costs of conventional and unconventional imaging modalities [2]. The combination of structural and molecular information is of great necessity to enable individualization of pulmonary pathologies and open novel avenues for advancing personalized medicine in the field of therapeutic diagnostics (theranostics), a companion diagnostic system where the molecular targeting probe is used to carry a dye- or gamma photon-based isotope to image target expression and also to carry a therapeutic compound to the very same target. In this review, we describe principal pulmonary diseases, clinical diagnosis issues, and molecular imaging tools currently available or being studied.

**Table 1:** Imaging modalities general characteristics

Modality	Spatial Resolution	Parameters imaged	Depth	Acquisition time	Cost per scan^a^ (US $)
PET	1-2 mm	Metabolism, immunology	No limit	minutes/h	$1000–$2000
SPECT	1-2 mm	Metabolism, immunology	No limit	minutes/h	$1000–$1500
MRI	<100 μm	Structure, metabolism	No limit	minutes/h	$800–$1200
Chest X-ray	<100 μm	Structure	No limit	minutes	$200–$400
CT	<100 μm	Structure	No limit	minutes	$600–$800

^a^Cost per scan may vary depending on the injected dose price. Adapted from [2]

### Lung cancer

Lung cancer is the leading cause of cancer-related death in men worldwide, and the second leading cause in women [3]. In the United States, lung cancer is the leading cause of cancer death in both men and women [3]. It is estimated that greater than 25% of cancer-related deaths in the United States in 2017 will be caused by lung cancer, and that it will kill more people than breast, colon, and prostate cancer combined [4]. Lung cancer has three main histologic categories: non-small cell lung cancer (NSCLC), small-cell lung cancer (SCLC), and neuroendocrine tumors (NET) [5]. NSCLC is most common, accounting for 85–90% of all lung cancers, and is made up of squamous cell carcinoma, adenocarcinoma, and large cell carcinoma. The main risk factor for the development of lung cancer is smoking. Other risk factors include exposure to air pollution, second-hand smoke, radiation (either radon decay or from medical X-rays), and asbestos [5]. Independent of type, early and accurate diagnosis of lung cancer is important to increase the likelihood of survival. Chest radiographs are the first line of conventional imaging used to detect abnormal areas in the lung but are limited in their capacity to detect cancer due to low-resolution and the two-dimensional nature of the image. Low-dose spiral CT is therefore the gold standard for lung cancer diagnosis and staging [5]. The National Lung Screening Trial found that patients at high-risk for lung cancer randomized to yearly CT scans had a 20% reduction in lung cancer mortality compared with patients randomized to yearly chest radiographs [6]. However, low-dose CT does have limitations, including a higher dose of radiation, more frequent detection of questionable lesions leading to potentially unnecessary biopsies or surgical intervention, and inability to detect chest wall invasion [7, 8]. Due to the limitations of low-dose CT and the fact that lung nodules are often found incidentally, patients with lung cancer would benefit from improved imaging modalities that can provide early detection, diagnosis, and appropriate staging.

Solitary pulmonary nodule (SPN) is the name given to a small isolated dense region observed on chest X-ray and CT scans. SPNs are sources of preoccupation since they can be of benign or malignant causes such as inflammation, infection or lung cancer [9]. PET/CT and its high detection sensitivity associated with molecularly targeted probes can permit further distinction. In this regard, [^18^F]-fluorodeoxyglucose ([^18^F]-FDG) is the most commonly use radiotracer in oncology. Its ability to mimic glucose reveals information about cellular glucose metabolism and allows discrimination of cell populations or tissues with high glucose metabolic rate, usually active proliferation, from a quiescent cell population. However, not all cancers are [^18^F]-FDG avid, leading to false negative interpretation. In addition, metastatic spread can be misinterpreted if the primary tumor showed initially low [^18^F]-FDG uptake. On the other hand, false positive scans can occur as inflammatory or infectious condition protagonists tend to have high metabolic rate, thus exhibiting high [^18^F]-FDG uptake. A more accurate differential diagnosis is necessary to improve the outcome of lung cancer patients via appropriate staging, treatment, and assessment of the lung disease.

A hexosamine pathway tracer, [^99m^Tc]-ethylenedicysteine-glucosamine ([^99m^Tc]-ECG), was evaluated in phase 1 and phase 2 clinical trials where investigators were able to discriminate inflammation from tumors in NSCLC patients [10]. Further studies have been performed in mesothelioma-bearing rats [11]. Here, [^99m^Tc]-ECG and [^68^Ga]-ECG were taken up by tumors at a lower extent than [^18^F]-FDG, but still enough to exhibit a positive signal in comparison to muscle on in vivo images 30 min post-injection, thus providing discriminatory information on the hexosamine pathway in such tumors.

3′-deoxy-3′-[^18^F]-fluorothymidine ([^18^F]-FLT), an analogue of thymidine that is incorporated in DNA during proliferation, has been developed as an imaging marker of proliferation. Recently a meta-analysis study comparing Ki67 index from biopsies of 1213 lung cancer patients to [^18^F]-FDG or [^18^F]-FLT standard uptake values revealed respective pooled Rho values of 0.45 and 0.65, indicating higher reliability for [^18^F]-FLT to assess lung cancer cell proliferation [12]. Artificial amino acid tracers such as O-[^18^F]-fluoromethyl-L-tyrosine (FMT) [13], O-[^18^F]-fluoropropyl-L-tyrosine (FPT), and O-(2-[^18^F]-fluoroethyl)-L-tyrosine (FET), have also been evaluated as amino acid metabolism and protein synthesis is enhanced in tumors [14]**.**

Angiogenesis is a phenomenon of great interest in oncology. The more a tumor is vascularized, the higher is the risk of metastasis [15–17]. Expression of vascular endothelial growth factor receptor type 2 (VEGFR-2) in tumors has been proportionally correlated to poor patient prognosis. Luo et al. developed a PET [^64^Cu]-labeled monoclonal antibody targeted to VEGFR-2 [18]. In this study, the investigators were able to successfully discriminate high versus low VEGFR-2 expression levels in a mouse model bearing human NSCLC tumors using [^64^Cu]-NOTA-ramucirumab. Such capability would allow effective monitoring of the efficacy of therapeutic treatments with angiogenesis inhibitors in lung cancer.

Hypoxia has been observed and characterized in many tumors. Hypoxic cells are phenotypically different compared to the non-hypoxic cells that might have been originally used to validate the efficacy of the treatment. Hypoxia is one of the main players that will determine therapy efficacy. Nitroimidazole derivatives have been used in the development of molecular imaging tracers. [^18^F]-fluoromisonidazole-1-(2-nitroimidazolyl)-2-hydroxy-3-fluoropropane (FMISO) and [^18^F]-fluoroazomycin-arabinofuranoside (FAZA) showed strong correlation with measurements of tumor oxygenation [19]. However, FAZA exhibited a better signal-to-background ratio than FMISO due to FAZA hydrophilicity inducing fast clearance in preclinical studies [20]. Clinically, little impact has been made by hypoxia imaging tracers in lung cancer mostly due to the lack of understanding of the mechanistic actions of these imaging agents [19].

Matrix metalloproteinases (MMPs) are key players in cancer progression and are known to be overexpressed in lung tumor tissue [21]. Fluorescent bioactivable probes such as MMPSense 680 showed potential to delineate tumor mass in mutant K-ras mice [22]. This study demonstrated the ability of MMPSense 680 to discriminate normal tissue from adenoma or adenocarcinoma via fluorescent molecular tomography. Unfortunately, due to the lack of tissue penetration depth, the translation of fluorescent probes to a non-invasive clinical application is not yet feasible. However, intraoperative molecular imaging using such probes can be of tremendous help for surgeons as it facilitates tumor delineation, thus confirming that complete removal of tumor residue during surgical resection is possible [23].

In lung cancer as in other cancers, conventional imaging provides evidence for the existence of small tissue abnormalities or lesions to help guide decision making regarding whether to further investigate for malignancy. Among the various imaging modalities used in nuclear medicine, PET is the closest modality to CT imaging in terms of size limits of detection. The primary clinical role of PET imaging is the detection of anomalous regions of [^18^F]-FDG uptake, which are often indicative of malignant lesions. The limits of detectability of tumors depend on many parameters including location of the tumor and imaging isotope. Modern clinical PET scanners have a resolution limit of 4 mm, corresponding to the detection of tumors with a volume of 0.2 ml (7 mm diameter) in 5:1 tumor-to-background ratio; whereas the minimum lesion size that can be measured with CT is about 3 mm [24]. Significant research efforts are underway to develop new detector materials, improved camera design, and new reconstructive algorithms in order to improve the limits of tumor detectability.

### Acute respiratory distress syndrome (ARDS)

ARDS is an acute, progressive inflammatory lung disease that affects 10% of all intensive care unit (ICU) patients worldwide [25, 26]. The pathophysiology of ARDS includes lung inflammation leading to increased permeability of the pulmonary vasculature, accumulation of pulmonary edema, increased lung weight, and a reduction in lung surface area participating in gas exchange [27]. ARDS is diagnosed clinically using the Berlin definition, which includes timing of onset, appearance of chest imaging, origin of edema, and oxygenation capacity. Timing of onset for ARDS is within 1 week of new or worsening respiratory symptoms or of a known respiratory insult. Common risk factors for the development of ARDS include trauma caused by aspiration, burns, blood transfusion and noncardiogenic shock. However, most ARDS cases are mainly caused by pneumonia and sepsis [25, 28].

Many different viral, bacterial, and fungal organisms cause respiratory infections [29]. Collectively, these infections are one of the most prevalent worldwide public health problems [30]. Although many community-acquired respiratory infections are short-lived and are successfully managed as an outpatient with little to no treatment, respiratory infections cause more morbidity and mortality in the United States than any other infection [29]. The mortality rate for respiratory infections has remained rather stagnant for the past 50 years [31]. Patients who acquire a respiratory infection and are elderly, immunocompromised, or have multiple comorbidities or baseline lung dysfunction, may require inpatient hospitalization, ICU admission, mechanical ventilation, and broad-spectrum antibiotics. Respiratory infections are diagnosed through a combination of a thorough history and physical exam, clinical indicators (leukocytosis, presence of microorganisms in sputum culture), and imaging.

Common imaging modalities useful in the diagnosis of respiratory infections are chest X-ray and CT [32]. Both chest X-rays and CT scans provide nonspecific, supporting evidence for the presence of a respiratory infection, but they do not allow for identification of the etiology of a specific infection or the involved immune cell types. This lack of clinically available imaging techniques that could help identify the specifics of an infection impacts the clinician’s ability to provide timely, targeted therapeutic interventions. The use of molecular imaging may allow for earlier identification of patients who are progressing towards ARDS. Additionally, the ability to image respiratory infections using molecular markers may provide important information regarding the likely etiology of the infection (i.e. bacterial versus viral). This improvement in diagnosis may allow for earlier, targeted antibiotic treatment and protection from overtreatment when it is not likely to be beneficial.

**Fig. 1:**
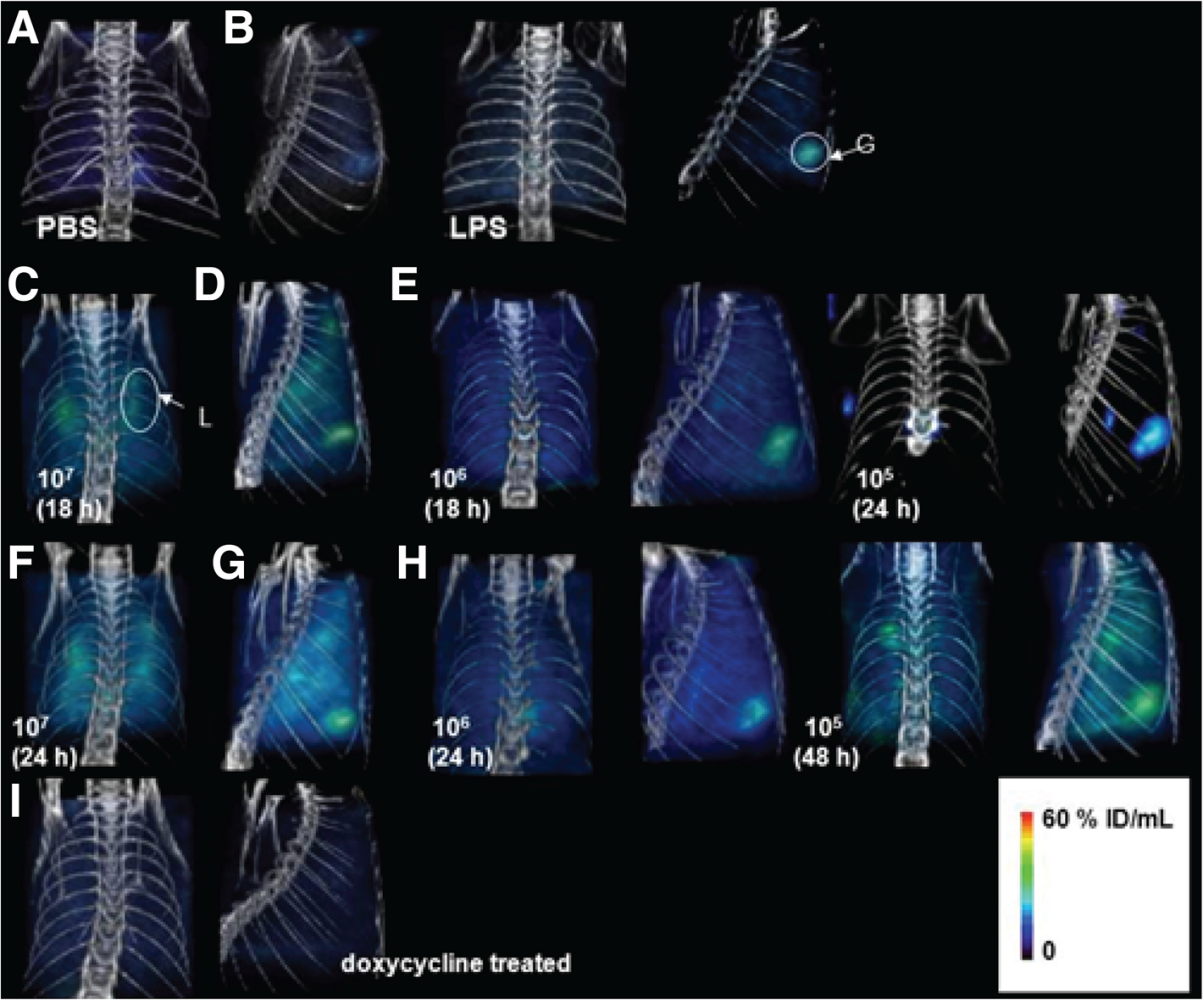
SPECT-CT imaging of lung inflammation and infection. Control (PBS) and LPS-treated (inflammation) mice were injected with 1 mCi (37 MBq) of [^125^I]-FIAU via the tail vein and SPECT-CT images were acquired at (**a**) 24 h after PBS administration (control), (**b**) 24 h after LPS administration. Four mice were imaged per group. All scans were performed two hours after injection of [^125^I]-FIAU. All images were adjusted to the same maximum signal threshold. Mice harboring lung infection were similarly injected with 1 mCi (37 MBq) of [^125^I]-FIAU with SPECT-CT images acquired at 18 h after infection with (**c**) 10^7^ CFU/mL, (**d**) 10^6^ CFU/mL, and at 24 h after infection with (**e**) 10^5^ CFU/mL of *E. coli RS218*. SPECT-CT images of mice imaged at 24 h after infection with (**f**) 10^7^ CFU/mL, (**g**) 10^6^ CFU/mL and at 48 h after infection with (**h**) 10^5^ CFU/mL of *E. coli RS218*. Four mice were imaged for each bacterial infecting dose. For monitoring antibiotic therapy, SPECT-CT images (**i**) of a mouse inoculated with 10^5^ CFU/mL *E. coli RS218* and with doxycycline for 10 days (5 mg/kg/day) were obtained. Four mice were imaged per group. A representative image is depicted for each group. G- gallbladder, S- stomach, L- Lung. (Reprinted from [36] with permission from e-Century Publishing Corporation)

Ex vivo administration of [^111^In]-labeled leukocytes are useful tools to detect infection [33]. In a cohort of 145 patients, administration of [^111^In]-WBCs showed a 94% sensitivity and 64% specificity for detecting pulmonary infections [34]. In addition, a 99% negative predictive value has been observed in 137 immuno-deficient patients without focal pulmonary activity [35]. The detection of bacterial load by targeting specific prokaryotic signatures is an alternative method that can be achieved using molecularly targeted probes. For instance, a radiolabeled nucleoside analog that is an exclusive substrate of bacterial thymidine kinase (TK), has been studied for this purpose. 2′-fluoro-2′-deoxy-1β-D-arabinofuranosyl-5-[^124/5^I]iodouracil ([^124/5^I]-FIAU) was injected in a mouse model of lipopolysaccharide (LPS)-induced inflammation as well as an *E. coli RS218* TK-positive infected mouse lung model (Fig. 1) [36]. The investigators were able to discriminate the inflammation state from infection state in the mice, reporting an [^124/5^I]-FIAU uptake 4 times greater (1.69 ± 0.40 vs. 7.14 ± 1.09 %ID/g, respectively). In vivo SPECT/CT images confirmed these results. However, the use of [^125^I] is usually not recommended for SPECT imaging purposes as the emitting energy is very low. Switching to PET would improve the quality of the images as well as quantitative analysis. Unfortunately, [^125^I]-FIAU PET has only been tested in humans for infection condition that is not lung related and did not show suitable results [37].

Bacterial ribosomal RNAs have also been studied as a bacterial marker. Chen et al. generated a [^99m^Tc]-radiolabeled 18-mer oligomer phosphorodiamidate morpholino sequence ([^99m^Tc]-MORF) that is complementary to a 16S rRNA exhibited by most bacteria [38]. The researchers found that [^99m^Tc]-MORF uptake was higher in mice with *Klebsiella pneumoniae* infection versus mice with sterile inflammation within 90 min. Qualitatively, radiotracer uptake was observed on SPECT/CT whole body images. However, the background appeared elevated.

Another interesting study by Jørgensen et al. evaluated inflammation and infection processes by comparing [^18^F]-FDG PET to uptake of cholinergic PET imaging agents such as [^18^F]-fluoroethoxybenzovesamicol ([^18^F]-FEOBV), a marker of vesicle cholinergic transporter, and [^11^C]-donepezil, a marker of acetylcholinesterase [39]. Using a murine model of *Staphylococcus aureus* infection as well as pigs displaying post-operative abscesses, the authors demonstrated a different accumulation kinetics compared to [^18^F]-FDG, suggesting that these two radiotracers image different aspects of immune response. Human data showed higher [^11^C]-donepezil activity at sites of chronic inflammation when compared to [^18^F]-FDG.

Radiolabeled antibiotics have been thought to be promising as they would be targeting specific bacteria items. Many antibiotics have been tested to this purpose [40]. Among them, ciprofloxacin, a member of the fluoroquinolone family, binds to gram-negative and gram-positive bacterial DNA gyrase. When radiolabeled with [^99m^Tc], ciprofloxacin penetrates mammalian cells and tissues but with low affinity and quick wash out. However, after a prolonged time, [^99m^Tc]-ciprofloxacin is retained at sites of infection allowing a target-to-background ratio favorable for imaging. High sensitivity (>90%) has been found in tuberculosis and other conditions reported from a large multicenter study [41]. However, a PET version of ciprofloxacin radiolabeled with [^18^F] showed rapid wash-in, wash-out and non-specific binding in the infected area, which depreciate the suitability of this radiotracer for infection sites [42]. Another fluoroquinolone-complex named norfloxacin dithiocarbamate (NFXDTC) has been well-characterized [43]. Zhang et al. radiolabeled NFXDTC with [^99m^Tc] and showed a 3.43±0.53% injected dose per gram (%ID/g) of infected area compared to 1.76±0.20%ID/g obtained with [^99m^Tc]-ciprofloxacin. However, lung and liver uptake are very elevated which prevent its use in related conditions. As many radiolabeled antibiotics have been or still are under investigation, none has yet demonstrated significant capacity to differentiate sterile inflammation from infection [40].

### Chronic lung disease

Chronic respiratory diseases, including chronic obstructive pulmonary disease (COPD) and asthma, cause significant morbidity and mortality worldwide, accounting for 4 million deaths annually [44, 45]. COPD, which includes emphysema and chronic bronchitis, can be suspected based on the presence of dyspnea, chronic cough, chronic sputum production, recurrent respiratory infections, and are associated with known risk factors such as exposure to tobacco smoke and occupational vapors [46, 47]. The gold standard for diagnosing COPD is the pulmonary function test (PFT), which uses spirometry to measure lung volumes. A post-bronchodilator ratio of the fraction of expired volume in one second to the forced vital capacity (FEV1/FVC) of less than 0.70 is indicative of persistent airflow limitation [46]. The mainstay of treatment for COPD is bronchodilation, using either short- or long-acting β2-adrenergic agonists or anticholinergic agents, depending on the severity of symptoms [46].

The role for conventional imaging modalities (chest x-rays and CT scans) in the diagnosis of COPD and other chronic lung diseases is to identify structural abnormalities (air trapping, airway septal thickening) and to rule out concomitant diseases that may be responsible for the patient’s respiratory symptoms. Unfortunately, these conventional modalities are limited in their ability to diagnose chronic lung diseases. Chest x-rays, while inexpensive and associated with low radiation exposure, are two-dimensional and have low resolution (Table 1). CT scans are significantly more valuable due to the increased spatial resolution and improved depth of penetration; however, they are expensive and require higher radiation doses (Table 1). Additionally, research has shown that the visual evaluation of CT scans has low reproducibility [48]. However, the use of densitometric evaluation improves the utility of CT scans in diagnosing chronic lung disease [49].

Asthma is another common chronic respiratory disease that can cause airflow obstruction and symptoms similar to COPD but differs in that asthma is reversible with proper treatment. Asthma is characterized by intermittent dyspnea, cough, and wheezing. Intermittent asthma is managed with short-acting β2-adrenergic agonists such as albuterol, while persistent asthma is treated with inhaled corticosteroids [50]. PFTs are used during the diagnostic workup of asthma, however results are often normal considering that asthma symptoms occur intermittently. Additionally, the use of bronchodilators during PFTs will demonstrate the reversible nature of the airway obstruction associated with asthma, which differs from the irreversible nature of COPD. Chest x-rays and CT scans have little role in the diagnosis of asthma and will usually not demonstrate any structural abnormalities. Asthma affects 7.5% of the population in the United States, with onset usually in childhood or as a young adult [50]. A family history of asthma, exposure to environmental toxins such as tobacco smoke, and low socioeconomic status are risk factors for the development of asthma [50]. The hallmark of airway inflammation in patients with asthma is allergen-specific IgE- and eosinophil-mediated neutrophil activation. Additional physiologic changes include mast cell degranulation and infiltration of T-helper lymphocytes, and is associated with elevated levels of IL-4, IL-5, and IL-13 [51–54].

Currently, planar lung scintigraphy is usually prescribed to visualize distribution of ventilation and perfusion in the lungs of patients with pulmonary embolism but also in cases of emphysema patients that undergo lung volume reduction surgery. However, use of 3-dimensional ventilation/perfusion (v/q) SPECT imaging methods have increased the diagnostic accuracy and expanded the application range to various pulmonary diseases. Technegas, ^99m^Tc-labelled carbon particles, has been tested as an aerosol in COPD patients [55]. With a moderate to strong correlation with spirometric lung function, Technegas has potential to provide valuable information for the diagnosis of COPD. However, more studies that link v/q SPECT to lung function need to be performed to fully validate the method.

**Fig. 2:**
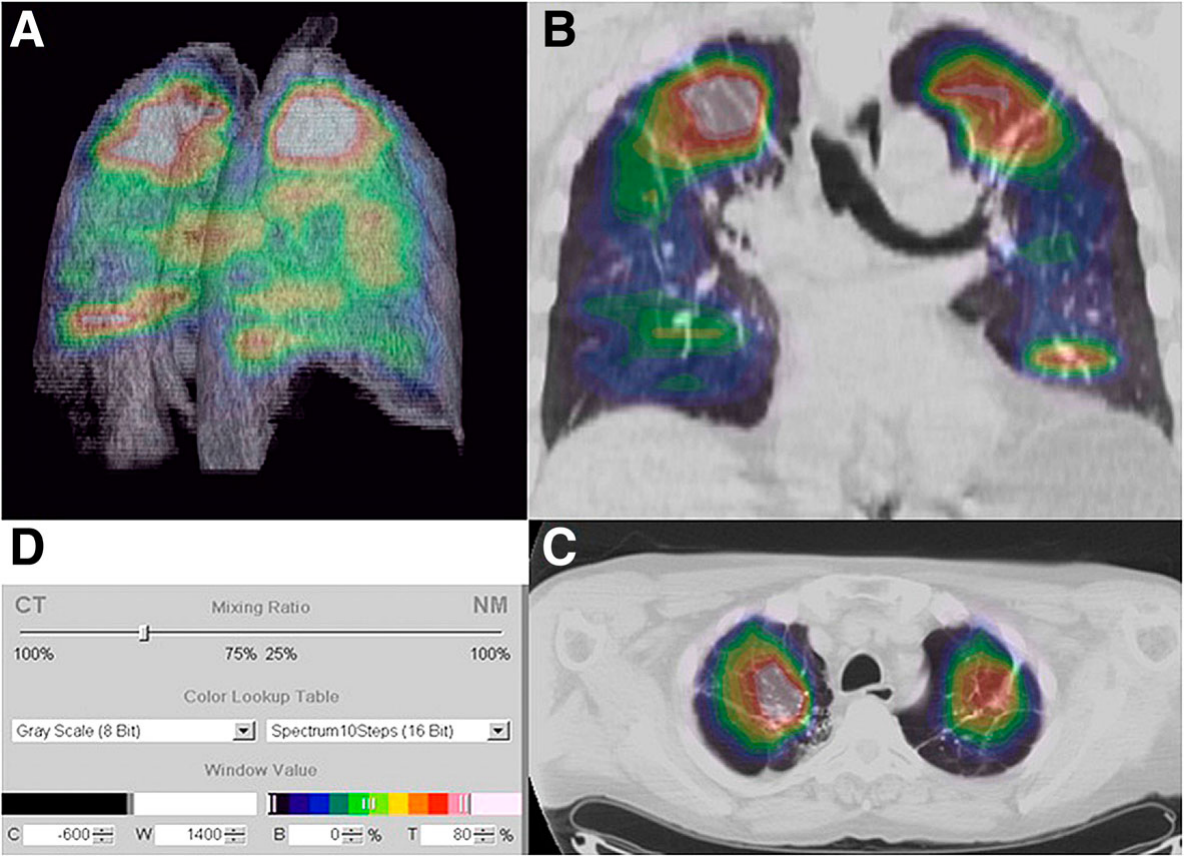
**a** Three-dimensional, (**b**) axial, and (**c**) coronal imaging illustrating the predominantly apical distribution of pulmonary ^18^fluorodeoxyglucose ([^18^F]-FDG) uptake in a patient with usual chronic obstructive pulmonary disease (COPD). **d** The color scale gives the spectrum applied to the full range of [^18^F]-FDG uptake, so that each color band represents a range of 10% of the maximum signal, with the maximum signal represented by *white* and the minimum signal by *black*. (Reprinted from [58] with permission of the American Thoracic Society. Copyright © 2017 American Thoracic Society)

Considering that conventional imaging modalities play a limited role in the diagnosis and management of chronic respiratory diseases, there exists a significant opportunity to develop cell type-specific molecular imaging strategies than can improve diagnosis, provide monitoring of treatment responsiveness, and limit invasive and often traumatic tissue biopsies. In addition, molecular imaging could have a role in quantifying with accuracy lung involvement in COPD, asthma and other chronic pulmonary diseases. The involvement of immune cells in the development of chronic lung diseases is an important topic, which was addressed in a study by Jones et al. that assessed neutrophilic infiltration and presence of macrophages in a small group of COPD and asthmatic patients [56]. Using PET imaging associated with [^18^F]-FDG to assess metabolism and [^11^C]-PK11195 as a marker of macrophages, the investigators found a 2.7- and 2.5-fold increase in [^18^F]-FDG uptake in COPD patients versus, respectively, asthmatic and healthy patients whereas [^11^C]-PK11195 tissue-to-plasma ratios were equally higher in asthma and COPD conditions compared to normal patients. The difference in [^18^F]-FDG uptake between COPD and asthma might come from a different state of neutrophil activation between the two conditions. [^18^F]-FDG avidity to neutrophil activation has also been demonstrated and quantified by PET imaging methods (Fig. 2) [57, 58].

**Fig. 3:**
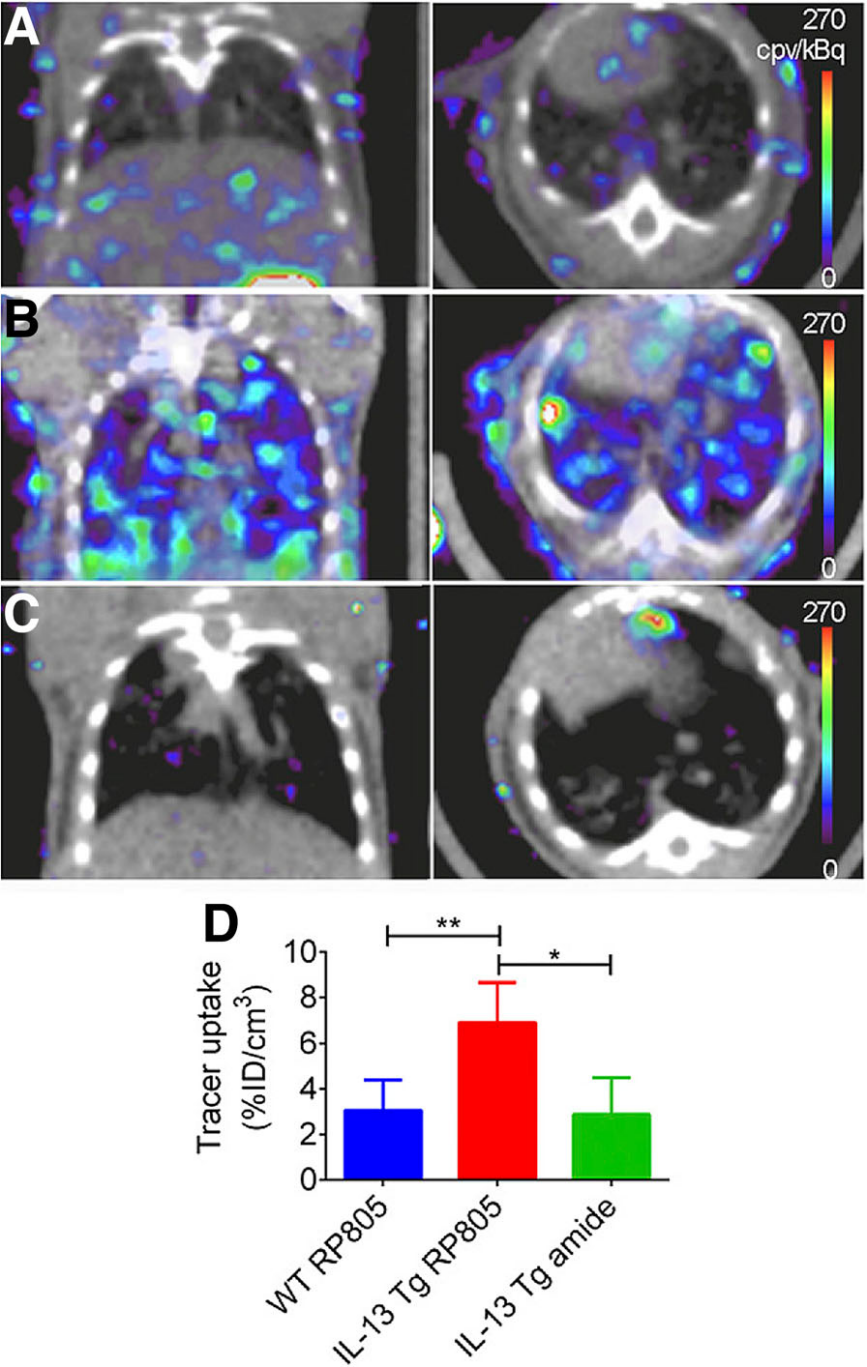
In vivo small-animal SPECT/CT imaging of MMP activation. **a–c** Examples of coronal (left) and transversal (right) views of fused small-animal SPECT/CT images of WT mice injected with RP805 (**a**) and CC10-IL-13 Tg mice injected with RP805 (**b**) or its control, amide analog tracer (**c**). **d** Small-animal SPECT–derived quantification tracer uptake in lungs. *N =* 5, 6, 13, and 5, respectively, for WT mice injected with RP805 and CC10-IL-13 Tg mice injected with RP805 or amide, control analog. *P, 0.01. **P, 0.001. cpv 5 counts per voxel; ID 5 injected dose. (This research was originally published in JNM [59]. © by the Society of Nuclear Medicine and Molecular Imaging, Inc.)

MMPs are known to play an important role in lung inflammation and remodeling. Using a transgenic mouse model that exclusively expresses IL-13 in lungs and an RP805 precursor [^99m^Tc]-labeled macrocyclic MMP-targeted tracer, Golestani et al. obtained a significant 2.5-fold increase in tracer uptake in these mice compared to wild-type animals via SPECT/CT imaging (Fig. 3) [59]. RP805 exhibited good correlation with CD68 expression (*r* = 0.70, *P* < 0.01). Other radiotracers such as [^68^Ga]-DOTANOC and [^111^In]-DTPA-A20FMDV2 may be useful for detection of fibroblast activity and AvB6-integrin expression in pulmonary fibrosis, respectively [60, 61].

### Lung transplant

#### Primary graft dysfunction (ischemia-reperfusion injury)

Lung transplantation is the standard of care for patients with end-stage pulmonary disease who have failed to respond to medical management [62]. Unfortunately, outcomes after lung transplantation are worse than outcomes for all other solid organs, with a 5-year mortality rate of 50% [62, 63]. Many different efforts are being explored that may improve the frequency and success rate of lung transplantations, including the use of ex vivo lung perfusion and increased utilization of organs donated after circulatory death (DCD) [64, 65]. In the acute postoperative period, ischemia-reperfusion injury (IRI) leading to primary graft dysfunction (PGD) is the major cause of early morbidity and mortality [66]. IRI is characterized by endothelial cell disruption, activation of alveolar macrophages and invariant natural killer T cells, neutrophil infiltration, and the release of pro-inflammatory cytokines and reactive oxygen species [67–70]. A clinical diagnosis of severe PGD (ratio of partial pressure of arterial oxygen to fraction of inspired oxygen [PaO_2_/FiO_2_] < 200 mmHg) is diagnosed in approximately 30% of patients within the first 72 h following lung transplantation. In addition to affecting short-term outcomes, PGD is also a significant risk factor for the development of bronchiolitis obliterans (also termed chronic lung allograft dysfunction), the leading cause of mortality 1 year following lung transplantation [71–73].

Currently, diagnosing PGD is limited to the use of conventional imaging methods such as chest radiography and CT scans, along with functional measurements of oxygenation capacity and compliance. The inability to rapidly and accurately diagnose PGD early after lung transplantation via non-invasive techniques restricts expanding the donor pool to include the use of marginal donor lungs (i.e. DCD lungs), which are associated with higher rates of IRI and PGD. Developing molecular imaging modalities to specifically target active inflammation and innate immune cell activation may allow for earlier diagnosis and earlier, targeted intervention.

Unfortunately, use of molecular imaging remains unexploited to address this clinical need. [^18^F]-FDG might be used as a general indication of inflammation in the lung, but knowing the activation status of specific innate immune cell populations at various stages would not only help to understand the physiopathology, but would also help to counteract any harmful effects caused by their activation or infiltration at the site of inflammation. Immune cell-specific molecular imaging tools are numerous. Although most of these have not been tested directly in pulmonary diseases, many have demonstrated preclinical promise in other inflammatory indications. For example, [^99m^Tc]-labeled-C2A domain of synaptotagmin I has been evaluated as a marker of necrosis and apoptosis in a rat model of myocardial IRI [74]. Dynamic SPECT images showed a clean hot spot in the wall of the left ventricle at 10 min post-injection that was retained over 2 h. The signal-to-vial myocardium and signal-to-lung ratios were 4.5 ± 0.2 and 8.2 ± 0.6, respectively. Myocardial apoptosis has also been studied in a rat IRI model using a PET imaging agent, [^18^F]WC-4-116, that targets caspase 3 activation [75]. The signal via myocardial autoradiography as well as via in vivo PET images had a 2-fold activity increase compared to myocardial area considered not at risk.

**Fig. 4:**
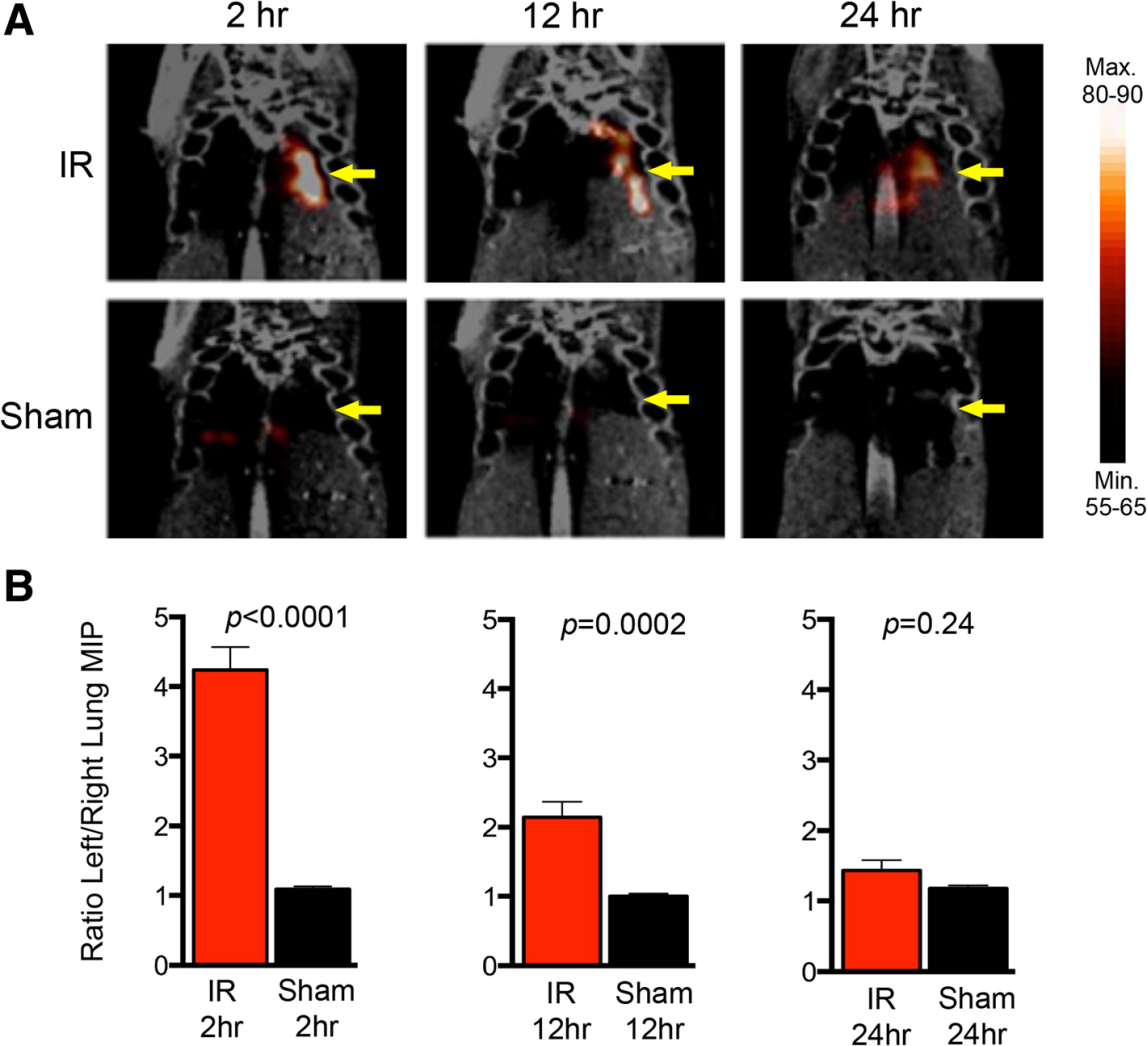
SPECT/CT imaging of lung ischemia-reperfusion injury (IRI) with [^99m^Tc]-cFLFLF. A murine model of IRI via left lung hilar occlusion was used in which lungs underwent sham surgery or 1 h of ischemia followed by reperfusion. SPECT/CT was performed after 2, 12, or 24 h of reperfusion after IR or sham surgery. Mice were injected with [^99m^Tc]-cFLFLF 2 h prior to imaging. **a** Representative SPECT/CT images at 2, 12, or 24 h of reperfusion. **b** Quantification of SPECT maximum intensity projections (MIP), reported as the ratio of left to right lung values (n=5-7/group), ±SEM

Recently, Liu et al. reported the use of a CCR2-binding peptide (64Cu-DOTA-ECL1i) for PET imaging of lung IRI in a murine lung transplant model [76]. This study, which targeted CCR2+ cells (monocytes), introduced the powerful potential of cell-specific molecular imaging techniques for noninvasive diagnosis of lung IRI. Our laboratory has begun to investigate molecular imaging methods to provide a more rapid and accurate diagnosis of lung IRI. As such, we have recently utilized [^99m^Tc]-cFLFLF, a PEGylated peptide ligand that binds formyl peptide receptor 1 (FPR1) on activated neutrophils, to perform SPECT/CT imaging in a murine model of left lung IRI [77]. Here, lung maximum intensity projections (MIP) were correlated with lung function after varying times of reperfusion. Left-to-right lung ratios of MIP and probe uptake were 3.8- and 2.8-fold higher, respectively, compared with sham animals (Fig. 4), which correlated with resolution of injury over time as measured by lung function and histologic presence of neutrophils. These results suggest that [^99m^Tc]-cFLFLF SPECT imaging may be a means to accurately provide quantifiable, noninvasive diagnosis of lung IRI as well as enable monitoring of the resolution of this injury over time.

### Graft rejection

In addition to IRI and PGD, allograft rejection is another serious complication following lung transplantation that contributes to poor outcomes. Cell-mediated (recognition of foreign human leukocyte antigens by recipient T lymphocytes) and antibody-mediated (binding of recipient antibodies to donor cell antigens) rejection can occur both in the short- and long-term following lung transplantation [78]. Diagnosing allograft rejection can be difficult due to variations in clinical presentation, with some patients being completely asymptomatic. Transbronchial biopsies are therefore required and are the gold standard for diagnosing rejection [78]. Given the inherent risks associated with transbronchial biopsies (bleeding, infection, pneumothorax), a noninvasive molecular imaging strategy using cell type- or antibody-specific labeling could provide a significant advancement in the diagnosis and surveillance of allograft rejection.

As previously mentioned for PGD, molecular imaging for acute or chronic lung rejection has not been widely studied. However, methods used to visualize cellular and molecular components of rejection such as cell membrane disruption, apoptosis and monitoring of immune cell activation in heart rejection may be applied to the lung [79]. In addition, a study by Chen et al. demonstrated that [^18^F]-FDG uptake was significantly increased in a mouse model of orthotopic lung transplantation and rejection [80]. These authors demonstrated that [^18^F]-FDG uptake occurred largely in CD8(+) T cells and was markedly attenuated following T cell depletion therapy.

## Conclusions

Despite efforts in the use [^18^F]-FDG PET imaging to detect inflammatory sites in the lung, its non-specific characteristic prevents any differential diagnosis of pulmonary disease or injury. When it comes to accurate diagnosis, staging, or post-treatment monitoring of disease, the use of molecular imaging would be a valuable tool to complement conventional imaging modalities. One of the biggest challenges ahead will be utilizing the optimal combination of imaging modality and molecular probe(s) in order to obtain an accurate and differential diagnosis. It is likely that accurate diagnoses of some pathologies will require the use of multiple molecular probes, especially in cases that involve numerous cell populations and complex or overlapping cell signaling pathways. For example, use of a neutrophil-specific probe, by itself, would not differentiate between various neutrophil-mediated inflammatory conditions in the lung such as infection, ischemia-reperfusion injury, antibody-mediated rejection, or ARDS. Although PET would provide high-resolution images, if needed, PET imaging is limited to a single radionuclide-labeled probe. However, combining imaging data with other clinical parameters will always greatly facilitate a more accurate diagnosis. One advantage of SPECT imaging is that multiple molecular probes (linked to different radionuclides) could be used to simultaneously monitor the status of various cell populations (or signaling pathways) in the lung to discriminate between overlapping pathologies such as infection, inflammation or rejection. However, a disadvantage of SPECT is its lower resolution versus PET.

Current molecular imaging methods under development, which allow visualization of specific cellular and molecular components of pulmonary disease including immune cell activation, show promise. However, thus far, the application of molecular imaging in pulmonary disease remains an under-explored field, and further studies are warranted in order to explore and develop novel cell-specific molecular probes and non-invasive imaging methods to provide differential diagnosis of lung disease. These future studies will undoubtedly reveal useful pulmonary imaging probes and imaging modalities.

## Abbreviations

ARDS: Acute respiratory distress syndrome
COPD: Chronic obstructive pulmonary disease
CT: Computed tomography
DCD: Donated after circulatory death
DOTA: Tetraazacyclododecane tetraacetic acid
DTPA: Diethylenetriamine pentaacetic acid
ECG: Ethylenedicysteine-glucosamine
FAZA: Fluoroazomycin-arabinofuranoside
FDG: Fluorodeoxyglucose
FEOBV: Fluoroethoxybenzovesamicol
FET: Fluoroethyl-L-tyrosine
FEV1: Fraction of expired volume in one second
FGFR-2: Vascular endothelial growth factor receptor type 2
FIAU: 2′-[(18)F]-fluoro-2′-deoxy-1-beta-D-arabinofuranosyl-5-iodouracil
FLT: Fluorothymidine
FMISO: Fluoromisonidazole-1-(2-nitroimidazolyl)-2-hydroxy-3-fluoropropane
FMP: Fluoromethyl-L-tyrosine
FPR1: Formyl peptide receptor 1
FPT: Fluoropropyl-L-tyrosine
FVC: Forced vital capacity
ICU: Intensive care unit
IgE: Immunoglobulin E
IL: Interleukin
IRI: Ischemia-reperfusion injury
LPS: Lipopolysaccharide
MIP: Maximum intensity projection
MMP: Matrix metalloproteinase
MORF: Phosphorodiamidate morpholinos
MRI: Magnetic resonance imaging
NET: Neuroendocrine tumors
NFXDTC: Norfloxacin dithiocarbamate
NSCLC: Non-small cell lung cancer
PBS: Phosphate Buffer Solution
PET: Positron emission tomography
PGD: Primary graft dysfunction
SCLC: Small-cell lung cancer
SPECT: Single photon emission computed tomography
SPN: Solitary pulmonary nodule
TK: Thymidine kinase

## References

[1] Anderson CJ, Lewis JS (2017). Current status and future challenges for molecular imaging. Philos Trans A Math Phys Eng Sci.

[2] Dimastromatteo J, Brentnall T, Kelly KA (2016). Imaging in pancreatic disease. Nat Rev Gastroenterol Hepatol.

[3] Torre LA, Siegel RL, Jemal A (2016). Lung cancer statistics. Adv Exp Med Biol.

[4] Siegel RL, Miller KD, Jemal A (2017). Cancer statistics, 2017. CA Cancer J Clin.

[5] Ambrosini V, Nicolini S, Caroli P, Nanni C, Massaro A, Marzola MC (2012). PET/CT imaging in different types of lung cancer: an overview. Eur J Radiol.

[6] Aberle DR, Adams AM, Berg CD, Black WC, Clapp JD, National Lung Screening Trial Research Team (2011). Reduced lung-cancer mortality with low-dose computed tomographic screening. NEJM.

[7] Carson J, Finley DJ (2011). Lung cancer staging: an overview of the new staging system and implications for radiographic clinical staging. Semin Roentgenol.

[8] Bunn PA (2012). Worldwide overview of the current status of lung cancer diagnosis and treatment. Arch Pathol Lab Med..

[9] Gould MK, Tang T, I-LA L, Lee J, Zheng C, Danforth KN (2015). Recent trends in the identification of incidental pulmonary nodules. Am J Respir Crit Care Med.

[10] Schechter NR, Erwin WD, Yang DJ, Kim EE, Munden RF, Forster K (2009). Radiation dosimetry and biodistribution of (99m)Tc-ethylene dicysteine-deoxyglucose in patients with non-small-cell lung cancer. Eur J Nucl Med Mol Imaging.

[11] Zhang YH, Bryant J, Kong FL, DF Y, Mendez R, Edmund Kim E (2012). Molecular imaging of mesothelioma with (99m)Tc-ECG and (68)Ga-ECG. J Biomed Biotechnol.

[12] Shen G, Ma H, Pang F, Ren P, Kuang A. Correlations of 18F–FDG and 18F–FLT uptake on PET with Ki-67 expression in patients with lung cancer: a meta-analysis. Acta Radiol. 2017;Jan 1:284185117706609 [Epub ahead of print]. 10.1177/0284185117706609 28475024

[13] Iwata R, Furumoto S, Pascali C, Bogni A, Ishiwata K (2003). Radiosynthesis ofO-[11C]methyl-L-tyrosine andO-[18F]Fluoromethyl-L-tyrosine as potential PET tracers for imaging amino acid transport. J Label Comp Radiopharm.

[14] Tsukada H, Sato K, Fukumoto D, Kakiuchi T (2006). Evaluation of D-isomers of O-18F-fluoromethyl, O-18F-fluoroethyl and O-18F-fluoropropyl tyrosine as tumour imaging agents in mice. Eur J Nucl Med Mol Imaging.

[15] Saharinen P, Tammela T, Karkkainen MJ, Alitalo K (2004). Lymphatic vasculature: development, molecular regulation and role in tumor metastasis and inflammation. Trends Immunol.

[16] Blood CH, Zetter BR (1990). Tumor interactions with the vasculature: angiogenesis and tumor metastasis. Biochim Biophys Acta.

[17] Sleeman JP, Thiele W (2009). Tumor metastasis and the lymphatic vasculature. Int J Cancer.

[18] Luo H, England CG, Graves SA, Sun H, Liu G, Nickles RJ (2016). PET imaging of VEGFR-2 expression in lung cancer with 64Cu-labeled Ramucirumab. J Nucl Med.

[19] Yip C, Blower PJ, Goh V, Landau DB, Cook GJ (2015). Molecular imaging of hypoxia in non-small-cell lung cancer. Eur J Nucl Med Mol Imaging.

[20] Chia K, Fleming IN, Blower PJ (2012). Hypoxia imaging with PET: which tracers and why?. Nucl Med Commun.

[21] Lim M, Jablons DM. Matrix metalloproteinase expression in lung cancer. Lung Cancer. 2002:349–56. 10.1385/1-59259-323-2:349 12415707

[22] Salaün M, Peng J, Hensley HH, Roder N, Flieder DB, Houlle-Crépin S (2015). MMP-13 in-vivo molecular imaging reveals early expression in lung adenocarcinoma. PLoS One.

[23] Keating JJ, Okusanya OT, De Jesus E, Judy R, Jiang J, Deshpande C (2016). Intraoperative molecular imaging of lung adenocarcinoma can identify residual tumor cells at the surgical margins. Mol Imaging Biol.

[24] Erdi YE (2012). Limits of tumor detectability in nuclear medicine and PET. Mol Imaging Radionucl Ther.

[25] Bellani G, Laffey JG, Pham T, Fan E, Brochard L, Esteban A (2016). Epidemiology, patterns of care, and mortality for patients with acute respiratory distress syndrome in intensive care units in 50 countries. JAMA.

[26] Pham T, Rubenfeld GD (2017). Fifty years of research in ARDS.The epidemiology of acute respiratory distress syndrome. A 50th birthday review. Am J Respir Crit Care Med.

[27] ARDS Definition Task Force, Ranieri VM, Rubenfeld GD, Thompson BT, Ferguson ND, Caldwell E, et al. Acute respiratory distress syndrome: the berlin definition. JAMA. 2012:2526–33. 10.1001/jama.2012.5669 22797452

[28] Alberti C, Brun-Buisson C, Burchardi H, Martin C, Goodman S, Artigas A (2002). Epidemiology of sepsis and infection in ICU patients from an international multicentre cohort study. Intensive Care Med.

[29] Mizgerd JP (2008). Acute lower respiratory tract infection. The New England journal of medicine. Massachusetts medical. Society.

[30] Mizgerd JP (2006). Lung infection—a public health priority. PLoS Med.

[31] Armstrong GL, Conn LA, Pinner RW (1999). Trends in infectious disease mortality in the United States during the 20th century. JAMA.

[32] Walker CM, Abbott GF, Greene RE, J-AO S, Vummidi D, Digumarthy SR (2014). Imaging pulmonary infection: classic signs and patterns. Am J Roentgenol.

[33] Schuster DM, Alazraki N (2002). Gallium and other agents in diseases of the lung. Semin Nucl Med.

[34] Segall GM, McDougall IR (1986). Diagnostic value of lung uptake of indium-111 oxine-labeled white blood cells. Am J Roentgenol.

[35] Love C, Tomas MB, Palestro CJ (2002). Pulmonary activity on labelled leukocyte images: patterns of uptake and their significance. Nucl Med Commun.

[36] Pullambhatla M, Tessier J, Beck G, Jedynak B, Wurthner JU, Pomper MG (2012). [(125)I]FIAU imaging in a preclinical model of lung infection: quantification of bacterial load. Am J Nucl Med Mol Imaging..

[37] Zhang XM, Zhang HH, McLeroth P, Berkowitz RD, Mont MA, Stabin MG (2016). [(124)I]FIAU: Human dosimetry and infection imaging in patients with suspected prosthetic joint infection. Nucl Med Biol.

[38] Chen L, Wang Y, Cheng D, Liu X, Dou S, Liu G (2013). (99m)Tc-MORF oligomers specific for bacterial ribosomal RNA as potential specific infection imaging agents. Bioorg Med Chem.

[39] Jørgensen NP, Alstrup AKO, Mortensen FV, Knudsen K, Jakobsen S, Madsen LB (2017). Cholinergic PET imaging in infections and inflammation using (11)C-donepezil and (18)F-FEOBV. Eur J Nucl Med Mol Imaging.

[40] Ferro-Flores G, Ocampo-Garcia BE, Melendez-Alafort L (2012). Development of specific radiopharmaceuticals for infection imaging by targeting infectious micro-organisms. Curr Pharm Des.

[41] Britton KE (2002). Imaging bacterial infection with 99mTc-ciprofloxacin (Infecton). J Clin Pathol.

[42] Langer O, Brunner M, Zeitlinger M, Ziegler S, Iler U M, Dobrozemsky G (2004). In vitro and in vivo evaluation of [18F]ciprofloxacin for the imaging of bacterial infections with PET. Eur J Nucl Med Mol Imaging.

[43] Zhang J, Zhang S, Guo H, Wang X (2010). Synthesis and biological evaluation of a novel 99mTc(CO)3 complex of ciprofloxacin dithiocarbamate as a potential agent to target infection. Bioorg Med Chem Lett.

[44] Milavetz G (2008). Global surveillance, prevention and control of chronic respiratory diseases: a comprehensive approach global surveillance, prevention and control of chronic respiratory diseases: a comprehensive approach;edited by BousquetJean and KhaltaevNikolai. J Pharm Technol.

[45] Savant AP, McColley SA (2017). Cystic fibrosis year in review 2016. Pediatr Pulmonol.

[46] Vogelmeier CF, Criner GJ, Martinez FJ, Anzueto A, Barnes PJ, Bourbeau J, et al. Global strategy for the diagnosis, management, and prevention of chronic obstructive lung disease 2017 report. GOLD executive summary. Am J Respir Crit Care Med. 2017:557–82. 10.1164/rccm.201701-0218PP 28128970

[47] Forey BA, Thornton AJ, Lee PN (2011). Systematic review with meta-analysis of the epidemiological evidence relating smoking to COPD, chronic bronchitis and emphysema. Pulm Med.

[48] Mascalchi M, Diciotti S, Sverzellati N, Camiciottoli G, Ciccotosto C, Falaschi F (2012). Low agreement of visual rating for detailed quantification of pulmonary emphysema in whole-lung CT. Acta Radiol.

[49] Mascalchi M, Camiciottoli G, Diciotti S (2017). Lung densitometry: why, how and when. J Thorac Dis.

[50] McCracken JL, Veeranki SP, Ameredes BT, Calhoun WJ (2017). Diagnosis and Management of Asthma in adults: a review. JAMA.

[51] James A, Mauad T, Abramson M, Green F (2012). Airway smooth muscle hypertrophy and hyperplasia in asthma. Am J Respir Crit Care Med.

[52] Lane SJ, Lee TH. Mast cell effector mechanisms. J Allergy Clin Immunol. 1996;98:S67–71–discussionS71–2. 10.1016/s0091-6749(96)80131-x 8939179

[53] Robinson DS, Bentley AM, Hartnell A, Kay AB, Durham SR (1993). Activated memory T helper cells in bronchoalveolar lavage fluid from patients with atopic asthma: relation to asthma symptoms, lung function, and bronchial responsiveness. Thorax.

[54] Rubin BK (2014). Secretion properties, clearance, and therapy in airway disease. Transl Respir Med.

[55] Jögi J, Ekberg M, Jonson B, Bozovic G, Bajc M (2011). Ventilation/perfusion SPECT in chronic obstructive pulmonary disease: an evaluation by reference to symptoms, spirometric lung function and emphysema, as assessed with HRCT. Eur J Nucl Med Mol Imaging.

[56] Jones HA, Marino PS, Shakur BH, Morrell NW (2003). In vivo assessment of lung inflammatory cell activity in patients with COPD and asthma. Eur Respir J.

[57] Jones HA, Cadwallader KA, White JF, Uddin M, Peters AM, Chilvers ER (2002). Dissociation between respiratory burst activity and deoxyglucose uptake in human neutrophil granulocytes: implications for interpretation of (18)F-FDG PET images. J Nucl Med.

[58] Subramanian DR, Jenkins L, Edgar R, Quraishi N, Stockley RA, Parr DG (2012). Assessment of pulmonary neutrophilic inflammation in emphysema by quantitative positron emission. Tomography.

[59] Golestani R, Razavian M, Ye Y, Zhang J, Jung JJ, Toczek J (2017). Matrix metalloproteinase-targeted imaging of lung inflammation and remodeling. J Nucl Med.

[60] Ambrosini V, Zompatori M, De Luca F, Antonia D, Allegri V, Nanni C (2010). 68Ga-DOTANOC PET/CT allows somatostatin receptor imaging in idiopathic pulmonary fibrosis: preliminary results. J Nucl Med.

[61] John AE, Luckett JC, Tatler AL, Awais RO, Desai A, Habgood A (2013). Preclinical SPECT/CT imaging of αvβ6 integrins for molecular stratification of idiopathic pulmonary fibrosis. J Nucl Med.

[62] Yusen RD, Edwards LB, Kucheryavaya AY, Benden C, Dipchand AI, Goldfarb SB (2015). The registry of the International Society for Heart and Lung Transplantation: thirty-second official adult lung and heart-lung transplantation report--2015; focus theme: early graft failure. J Heart Lung Transplant.

[63] Valapour M, Paulson K, Smith JM, Hertz MI, Skeans MA, Heubner BM (2013). OPTN/SRTR 2011 Annual Data Report: lung. Am J Transplant.

[64] Charles EJ, Huerter ME, Wagner CE, Sharma AK, Zhao Y, Stoler MH (2016). Donation after circulatory death lungs transplantable up to six hours after ex vivo lung perfusion. Ann Thorac Surg.

[65] Cypel M, Levvey B, Van Raemdonck D, Erasmus M, Dark J, Love R (2015). International Society for Heart and Lung Transplantation donation after circulatory death registry report. J Heart Lung Transplant.

[66] Porteous MK, Diamond JM, Christie JD (2015). Primary graft dysfunction: lessons learned about the first 72 h after lung transplantation. Curr Opin Organ Transplant.

[67] Laubach VE, Sharma AK (2016). Mechanisms of lung ischemia-reperfusion injury. Curr Opin Organ Transplant..

[68] Sharma AK, Lapar DJ, Zhao Y, Li L, Lau CL, Kron IL (2011). Natural killer T cell-derived IL-17 mediates lung ischemia-reperfusion injury. Am J Respir Crit Care Med.

[69] Sharma AK, Fernandez LG, Awad AS, Kron IL, Laubach VE (2007). Proinflammatory response of alveolar epithelial cells is enhanced by alveolar macrophage-produced TNF-alpha during pulmonary ischemia-reperfusion injury. Am J Physiol Lung Cell Mol Physiol.

[70] Fiser SM, Tribble CG, Long SM, Kaza AK, Cope JT, Laubach VE (2001). Lung transplant reperfusion injury involves pulmonary macrophages and circulating leukocytes in a biphasic response. J Thorac Cardiovasc Surg.

[71] Fiser SM, Tribble CG, Long SM, Kaza AK, Kern JA, Jones DR (2002). Ischemia-reperfusion injury after lung transplantation increases risk of late bronchiolitis obliterans syndrome. Ann Thorac Surg.

[72] Royer P-J, Olivera-Botello G, Koutsokera A, Aubert J-D, Bernasconi E, Tissot A (2016). Chronic lung allograft dysfunction: a systematic review of mechanisms. Transplantation.

[73] Thabut G, Mal H, Cerrina J, Dartevelle P, Dromer C, Velly J-F (2005). Graft ischemic time and outcome of lung transplantation: a multicenter analysis. Am J Respir Crit Care Med.

[74] Liu Z, Zhao M, Zhu X, Furenlid LR, Chen Y-C, Barrett HH (2007). Vivo dynamic imaging of myocardial cell death using 99mTc-labeled C2A domain of synaptotagmin I in a rat model of ischemia and reperfusion. Nucl Med Biol.

[75] Thukkani AK, Shoghi KI, Zhou D, Xu J, Chu W, Novak E (2016). PET imaging of in vivo caspase-3/7 activity following myocardial ischemia-reperfusion injury with the radiolabeled isatin sulfonamide analogue [(18)F]WC-4-116. Am J Nucl Med Mol Imaging.

[76] Liu Y, Li W, Luehmann HP, Zhao Y, Detering L, Sultan DH, et al. Noninvasive imaging of CCR2(+) cells in ischemia-reperfusion injury after lung transplantation. Am J Transplant. 2016;16:3016–23. 10.1111/ajt.13907 PMC5143208 27273836

[77] Laubach VE, Charles EJ, Cordia MD, Sharma AK, Mehaffey JH, Zhang Y, et al. Use of a novel formyl peptide receptor ligand and noninvasive SPECT imaging to diagnose and monitor ischemia-reperfusion injury after lung transplantation. Am J Respir Crit Care Med. 2017;195(Meeting Abstracts):A7617.

[78] Roden AC, Aisner DL, Allen TC, Aubry MC, Barrios RJ, Beasley MB (2017). Diagnosis of acute cellular rejection and antibody-mediated rejection on lung transplant biopsies: a perspective from members of the pulmonary pathology society. Arch Pathol Lab Med.

[79] Miller CA, Fildes JE, Ray SG, Doran H, Yonan N, Williams SG (2013). Non-invasive approaches for the diagnosis of acute cardiac allograft rejection. Heart.

[80] Chen DL, Wang X, Yamamoto S, Carpenter D, Engle JT, LI W (2013). Increased T cell glucose uptake reflects acute rejection in lung grafts. Am J Transplant.

